# Spontaneous Hemopneumothorax: A Rare but Life-Threatening Emergency

**DOI:** 10.7759/cureus.84641

**Published:** 2025-05-22

**Authors:** Michelle Sahagian, M. Sulaiman Ahmad, Samantha S Colon, Rocco Lafaro

**Affiliations:** 1 General Surgery, St. Barnabas Hospital (SBH) Health System, New York City, USA; 2 General Surgery, City University of New York (CUNY) School of Medicine, New York City, USA; 3 Cardiothoracic Surgery, St. Barnabas Hospital (SBH) Health System, New York City, USA

**Keywords:** emergency thoracotomy, hemopneumothorax, hemopneumothorax in young male, pleuritic chest pain, shortness of breath, spontaneous hemopneumothorax, spontaneous pneumothorax, tube thoracostomy

## Abstract

Spontaneous hemopneumothorax is rarely seen, but it is a surgical emergency. This case describes a young male who presented with chest pain and was found to have spontaneous hemopneumothorax. The patient was treated with chest tube insertion and emergent thoracotomy, as well as ligation of the suspected source of bleeding and resection of the affected lobe. The patient was discharged on postoperative day four and did not present with any issues or complications postoperatively. It is important to recognize and treat spontaneous hemopneumothorax promptly and appropriately with initial stabilization and then definitive surgical management in order to avoid morbidity and mortality. This case demonstrates a situation in which the patient was treated appropriately and avoided significant complications, and highlights the importance of future research into the contributing factors and pathophysiology of spontaneous hemopneumothorax.

## Introduction

Spontaneous hemopneumothorax (SHP) is a rare and potentially life-threatening condition characterized by the simultaneous presence of air and blood within the pleural cavity, occurring without antecedent trauma or iatrogenic intervention. The incidence of SHP is relatively low, accounting for approximately 1% to 12% of all spontaneous pneumothorax cases, with a notable predilection for young, tall, and slender males [1]. The pathophysiologic mechanisms underlying SHP are not entirely understood but are often associated with conditions such as tuberculosis or necrotizing pneumonia, which can lead to the rupture of vascularized adhesions or small blood vessels within subpleural blebs and bullae [2-5].

Clinically, SHP presents with the sudden onset of pleuritic chest pain, dyspnea, and signs of hemodynamic instability, such as hypotension and tachycardia. Prompt recognition and management are crucial to prevent severe complications, including hemorrhagic shock and respiratory failure. Imaging studies, particularly chest radiography and computed tomography (CT) scans, play a pivotal role in the diagnosis, revealing the coexistence of air and fluid in the pleural space.

Initial management involves stabilizing the patient's respiratory and circulatory status, commonly through the insertion of a chest tube to evacuate air and blood, which allows for lung re-expansion. However, definitive treatment often requires surgical intervention, including video-assisted thoracoscopic surgery (VATS). This study demonstrates the use of thoracotomy for emergent intervention due to continuous bleeding.

This case report aims to contribute to the limited body of knowledge on SHP by detailing a unique presentation and management of the condition, with an emphasis on the importance of early diagnosis and intervention.

## Case presentation

A 30-year-old male with no known medical history presented in the emergency room with a 12-hour history of severe left-sided pleuritic chest pain that began suddenly after coughing. The patient reported pain that worsened with deep breathing and was unrelieved since the onset. He also reported shortness of breath, chest tightness, and nausea. There was no past medical history, with social history significant for marijuana use. The patient denied a history of similar symptoms, prior trauma, or surgery. On arrival, his blood pressure was 126/86 mmHg, heart rate was 68 beats/minute, respiratory rate was 18 breaths/minute with an O₂ saturation of 100%, and body temperature was 36.6℃. The patient was not in acute distress.

**Figure 1:**
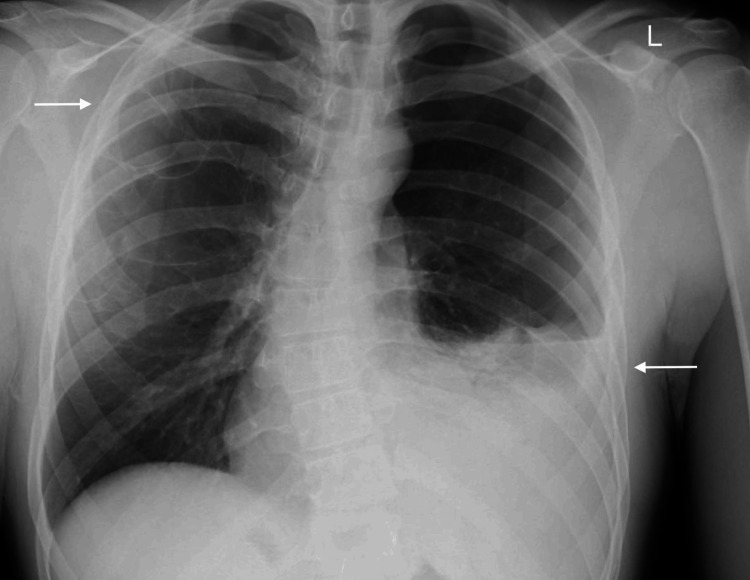
Chest X-ray showing left base effusion (lower arrow) with apical cystic bullae (upper arrow)

**Figure 2:**
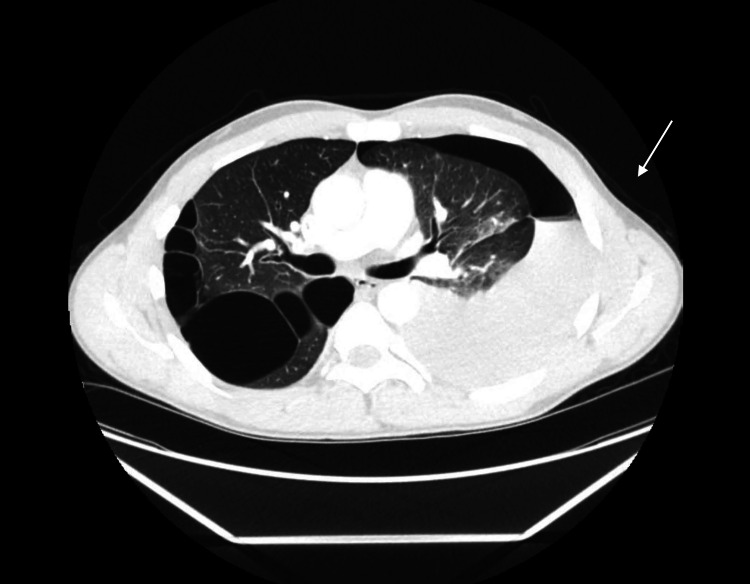
CT showing large left hemopneumothorax with active extravasation of contrast

A physical exam revealed decreased breath sounds on the left. Based on these findings, a pneumothorax was suspected, and an X-ray of the chest was obtained (Figure 1). Chest X-ray showed a left base effusion and cystic bullae in the apex of the right lung. A CT scan with IV contrast was then performed, which showed a large left hemopneumothorax with active extravasation of contrast material in the superior aspect of the pleural effusion (Figure 2).

After imaging was reviewed by the thoracic surgical team, a left pleural tube was placed for hydropneumothorax, which immediately drained 2 L of blood. Due to the amount of output, the patient was taken to the operating room for an emergent left thoracotomy.

**Figure 3:**
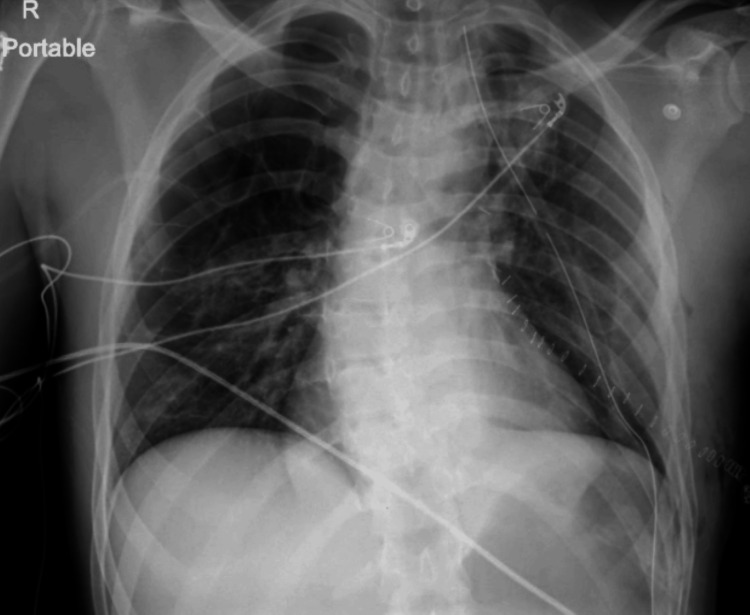
Postoperative chest X-ray with adequate lung expansion

Upon thoracotomy, active bleeding and clots were observed along with multiple large bullae in the left lung. The primary source of bleeding appeared to be a mediastinal arterial vessel, which was subsequently ligated. A segment resection of the left upper lobe apex was performed in conjunction with a bleb wedge resection of the lower lobe. The patient remained stable throughout the procedure and postoperatively, with minimal serosanguineous drainage from the chest tube. A chest X-ray was performed postoperatively to confirm lung expansion (Figure 3). 

The chest tube was removed on postoperative day four. The patient remained stable and was discharged later in the day. Sixteen days after discharge on postoperative day 20, the patient returned to the clinic for a follow-up appointment. At this time, he reported that he was breathing well and had no pain or complaints. During this visit, surgical staples were removed, and the patient was instructed to return to the clinic in four weeks; however was lost to follow-up.

## Discussion

SHP is an uncommon yet critical condition, defined by the concurrent presence of hemothorax and pneumothorax without prior trauma or medical intervention. The etiology of SHP remains largely idiopathic, though it is frequently linked to the rupture of vascularized pleural adhesions or small vessels in subpleural blebs and bullae [2-4]. This pathophysiology underscores the importance of a high index of suspicion in patients presenting with acute thoracic symptoms.

The demographic profile of SHP patients typically includes young, tall, and slender males, with studies suggesting an incidence rate of 1% to 12% among spontaneous pneumothorax cases [1]. Clinical presentations include sudden onset pleuritic chest pain, dyspnea, and symptoms of hemodynamic instability such as hypotension and tachycardia. These presentations necessitate immediate diagnostic imaging, primarily chest X-ray and CT scans, which reveal the coexistence of air and fluid in the pleural cavity. In this case, the cause of the bullae was unknown. This patient had no known medical history and no known history of connective tissue disorder. Further workup may have revealed an underlying cause, and further effort should continue to be made to encourage the patient to follow up.

Management of SHP requires prompt and effective intervention. Initial stabilization often involves the insertion of a chest tube to evacuate air and blood in the pleural cavity, thereby re-expanding the lung and alleviating respiratory distress. If a patient is hemodynamically stable, a tube thoracostomy is often the chosen intervention [6]. The volume of blood evacuated is a critical indicator of the severity of hemorrhage and guides further management. Definitive treatment typically involves surgical intervention, such as thoracotomy, to address ongoing bleeding and prevent recurrence.

The prognosis of SHP is contingent upon the promptness and effectiveness of the initial management. Delayed diagnosis and intervention can lead to significant morbidity and mortality due to complications such as hemorrhagic shock, persistent air leak, or empyema [7]. Hence, clinicians must maintain a high index of suspicion for SHP in young, otherwise healthy individuals presenting with acute chest pain and dyspnea.

## Conclusions

This case report highlights the importance of early recognition and timely surgical intervention in the management of SHP. Prompt identification and diagnosis were essential for appropriate resuscitation and surgical intervention in this case. While this patient recovered without any further issues, further research is needed. Identifying contributing risk factors and the pathophysiology of SHP is crucial. A deeper understanding of these factors will lead to improved diagnostic and treatment strategies, which will allow for the reduction of morbidity and mortality in affected patients.
